# Health Care Providers’ Experiences and Perspectives on the Use of Electronic Health Record System-ULTRA for Maternal and Perinatal Care in Kilimanjaro, Tanzania: Qualitative Study

**DOI:** 10.2196/90400

**Published:** 2026-11-06

**Authors:** Gaudensia A Olomi, Rachel Manongi, Karen Yeates, Charles Makasi, Victor W Katiti, Simon Woodworth, Ali S Khashan, Blandina T Mmbaga

**Affiliations:** 1 School of Public Health, KCMC University, PO Box 2240, Moshi, United Republic of Tanzania, 1 756831210; 2 Health Department, Kilimanjaro Regional Administrative Secretary's Office, Moshi, United Republic of Tanzania; 3 Kilimanjaro Clinical Research Institution, Moshi, United Republic of Tanzania; 4 Department of Medicine, Queen's University, Kingston, ON, Canada; 5 School of Medicine, KCMC University, Moshi, United Republic of Tanzania; 6 Research Station, National Institute for Medical Research, Kilosa, Morogoro Region, United Republic of Tanzania; 7 INFANT Research Centre, University College Cork, Cork, Ireland; 8 Cork University Business School, University College Cork, Cork, Ireland; 9 School of Public Health, University College Cork, Cork, Ireland; 10 Pediatric and Child Health Department, Kilimanjaro Christian Medical Centre, Moshi, United Republic of Tanzania

**Keywords:** electronic health record, digital health, maternal, perinatal, experience, barriers, facilitators, health care providers

## Abstract

**Background:**

Sub-Saharan Africa faces challenges with collecting maternal and perinatal data because of poor documentation in paper-based systems and inadequate infrastructure for digital utilization. The electronic health record system-ULTRA (EHRS-ULTRA) was developed and piloted in the Kilimanjaro region from October 2022 to August 2024 to improve data management.

**Objective:**

This qualitative study aimed to explore health care providers’ perspectives and experiences of using EHRS-ULTRA in maternal and perinatal health care.

**Methods:**

The study used a qualitative, exploratory, descriptive design to examine health care providers’ experiences using the EHRS-ULTRA in Kilimanjaro, Tanzania. Data were collected through key informant interviews and focus group discussions. Participants were purposively selected from 2 districts representing urban (Moshi Municipal) and rural (Moshi District) areas, including reproductive and child health coordinators; facility managers; and health care professionals from the antenatal care, labor and delivery, and postnatal care departments. A total of 35 participants took part, including 29 health care providers and 6 health managers. The data were analyzed through reflexive thematic analysis.

**Results:**

The EHRS-ULTRA was perceived by study participants to improve access to patients’ clinical information, continuity of care, quality of clinical decision-making, and quality of documentation for maternal and perinatal health services. Real-time notifications and referral tracking were reported to support timely clinical responses and leadership oversight. Key success factors for future adoption included leadership engagement, supportive supervision, and alignment of their existing Mfumo wa Taarifa za Uendeshaji wa Huduma za Afya (MTUHA) paper records system workflow. However, widespread challenges with unstable electricity, lack of reliable internet service, limited number of digital devices, and variability in digital literacy skills were perceived as the primary barriers to successful implementation and scaling of the system.

**Conclusions:**

Based on users’ experiences with the EHRS-ULTRA, health care professionals perceived EHRS-ULTRA as complementing existing paper-based documentation systems by improving information access, continuity of care, referral tracking, and the timeliness of clinical decisions about maternal and perinatal health care. EHRS-ULTRA demonstrated considerable potential to strengthen maternal and perinatal care; however, broader implementation and scale-up will require improvements in infrastructure, workforce readiness, and overall health system preparedness.

## Introduction

In resource-constrained countries, particularly in sub-Saharan Africa (SSA), maternal and perinatal data collection requires considerable effort because of the reliance on paper-based health management information systems, where health information is often poorly documented [1,2]. Consequently, decisions are often based on estimates derived from limited and poor-quality data, creating challenges for the implementation and evaluation of interventions [3]. Where high-quality data are available in paper-based medical charts, these records constitute a massive but underused source of maternal and perinatal data [4,5].

The current system, known as Mfumo wa Taarifa za Uendeshaji wa Huduma za Afya (MTUHA), Tanzania’s paper-based health management information system, faces challenges including poor data quality, incomplete documentation, delayed reporting, and nonstandardized terminology [6,7]. These shortcomings make data analysis and interpretation difficult, resulting in inconsistent information and delays in report generation [7]. Paper-based records are mostly shared and used in an aggregated form at all levels for clinical administration, program management, and strategic clinical decision-making. Their limitations include limited longitudinal patient tracking, difficulty retrieving records, limited utility for research, lack of data linkages between health facilities, and inadequate monitoring to support patient-centered care [3,8-10]. Digital health solutions, such as electronic health record systems (EHRSs), are increasingly being adopted to bridge existing gaps in health information systems, improve data accuracy, and support better clinical decision-making [11,12]. They facilitate faster access to patient information, thereby improving provider communication and care quality [13]. Additionally, digital health technologies are crucial for strengthening health systems and service delivery in low- and middle-income countries (LMIC), as emphasized by the World Health Organization (WHO). The WHO’s Global Strategy on digital health 2020‐2025 advocates for interoperable, user-centered systems to support continuity of care and clinical decision-making [14-16]. However, there is limited evidence on end user experiences with EHRSs in maternal and perinatal care within low-resource settings.

We recently conducted a systematic review on mobile health (mHealth) decision-making tools for maternal and neonatal health care, which identified challenges regarding usability, interoperability, workflow integration, and acceptance by health care providers throughout SSA [17]. Although many EHRSs have the potential to improve health care and record management, LMIC, including Tanzania, face challenges in adopting EHRSs because of competing health care priorities, limited resources, infrastructure limitations, and the health care providers’ perceptions of digital systems [3,18,19]. Although EHRSs aim to improve the efficiency and continuity of care, studies have also reported increased workloads for clinicians following implementation [20-23]. In Ghana, health professionals recognize the benefits of EHRSs, although unstable infrastructure and increased workload associated with electronic data entry and parallel documentation remain significant obstacles [11,12]. These challenges are further compounded by poorly designed and overly complex EHRS interfaces, which contribute to increased time spent using the system and clinician burnout [24].

To address these gaps in data quality and accessibility, valid and reliable maternal and perinatal health data are urgently needed to guide health care delivery in Tanzania [25]. Therefore, digital health innovations such as the EHRS-ULTRA have been introduced in Tanzania. The system was developed and piloted in the Kilimanjaro region between October 2022 and August 2024 to enhance maternal and perinatal care and data management. The EHRS-ULTRA was developed within the District Health Information System 2 (DHIS2) platform [26], an Android-based, open-source system that allows online and offline data collection and can be adapted for use across different levels of health care facilities. EHRS-ULTRA aligns with the WHO’s framework for digital health interventions, including client-level records and health management information systems supporting maternal and perinatal care. The system modifies the way data are currently recorded, covering antenatal care visits, labor and delivery, postnatal care, and routine child visit registers. EHRS-ULTRA aims to enhance maternal and perinatal health data recording and facilitate digital documentation within current health system workflows, intending to eventually replace paper-based MTUHA documentation and integrate with existing digital systems. During its pilot phase, it functioned concurrently with the mandatory MTUHA system due to national reporting requirements. Research is needed to understand how end users’ experiences can inform the implementation and refinement of emerging EHRS platform in their respective contexts.

Therefore, this study aimed to explore health care providers’ experiences and perceptions of using EHRS-ULTRA for maternal and perinatal care, including its perceived value, implementation challenges, and implications for future implementation and scale-up within the context of the WHO recommendations on digital health.

## Methods

### Study Design

A qualitative exploratory descriptive design was used to explore health care providers’ experiences and perceptions of using EHRS-ULTRA for maternal and perinatal care, including its perceived value, implementation challenges, and implications for future implementation and scale-up. This design was selected to explore health care providers’ early implementation experiences, including their perceptions of system usability and acceptability within routine clinical workflows. Data collection was conducted between June 2024 and July 2024 through focus group discussions (FGDs) and key informant interviews (KIIs).

### Study Area

The study was conducted in the Kilimanjaro region of northern Tanzania within the EHRS-ULTRA parent study (“EHRS-ULTRA: An Electronic Birth Registry for the Kilimanjaro Region: System Development and Pilot Implementation Study” [27]). The EHRS-ULTRA project introduced a phased pilot of EHRS-ULTRA beginning at the dispensary level, the lowest unit of health care facility delivery in Tanzania, and expanded to health centers (primary level of health care facility) and a regional referral hospital (secondary level of health care facility).

The pilot was carried out in 6 health facilities located in 2 districts of the Kilimanjaro region: Himo Cogi and Chekereni Dispensaries were included in the Moshi District Council, which represent rural areas, and Msaranga Dispensary, Pasua Health Center, Majengo Health Center, and Mawenzi Referral Regional Hospital in the Moshi Municipal Council were included to represent urban communities.

Municipal councils in Tanzania are urban-based with numerous health services and patients, while district councils are rural with fewer health services. In the context of this research, we did not examine whether the differences in administration between municipal and district councils affected the implementation or uptake of EHRS-ULTRA.

### Context for the Development of EHRS-ULTRA and Its Implementation

The development of the EHRS-ULTRA system is part of the broader EHRS-ULTRA project aimed at improving maternal and perinatal data management and continuity of care in the Kilimanjaro region of Tanzania. This system was collaboratively created by software developers, researchers, and health service stakeholders at regional, council, and health facility levels based on an adapted DHIS2 platform. The health care providers from health care facilities and health managers were involved in the iterative refinement process to ensure that the system was aligned with existing maternal and perinatal care workflows and the MTUHA documentation requirements.

A phased pilot implementation in selected health care facilities was conducted, operating alongside the existing paper-based MTUHA system. Health care providers received initial training and ongoing technical support during this period. A flowchart showing the EHRS-ULTRA platform workflow and implementation process can be found in Multimedia Appendix 1, and an outline of reporting and supervision in the health system is provided in Multimedia Appendix 2. Implementation of the EHRS-ULTRA system has been comprehensively documented and will be reported in a separate implementation study paper.

### Study Participants

The study participants included health care workers from the antenatal care, labor and delivery, and postnatal care departments, as well as facility managers (facility in-charges) and reproductive and child health teams from the council health management team, town council health management team, and regional health management team. Eligible participants were those who had received training on EHRS-ULTRA and were actively involved in its implementation or use during the pilot phase.

### Recruitment Procedures

Participants were recruited purposively from the 6 participating health facilities and relevant health management offices. Recruitment was facilitated by facility managers and relevant departmental supervisors who helped identify eligible participants through their direct involvement with EHRS-ULTRA. The research team then approached selected individuals in person, provided information about the purpose of the study, explained study procedures, and invited interested individuals to voluntarily participate. There were no eligible participants approached who declined participation or withdrew from the study after enrollment.

The principal investigator (GAO), a female public health researcher and PhD candidate who has both training and experience conducting qualitative research as well as working within the maternal health system, conducted all KIIs and moderated the FGDs as part of this project. In addition, the notetaker (VWK), a male PhD candidate and social worker, had previous experience collecting qualitative data as well as performing qualitative data analyses. Although some study participants were already familiar with the study team from participation in the EHRS-ULTRA implementation activities, there were no direct relationships of a supervisory nature between the study investigators and participants. Throughout the data collection and data analysis phases, the research team engaged in ongoing reflexive discussions to critically examine how their disciplinary backgrounds, professional experiences, and perspectives shaped data interpretation throughout the analytic process.

### Data Collection Tools

Semistructured interview guides were used for both FDGs and KIIs. The guides explored participants’ experiences and perceptions of the use of EHRS-ULTRA, including usability, data management, referral support, and notification of maternal and perinatal danger signs.

The guides were developed through a literature review, refined through expert consultation, and piloted with 2 participants working at similar levels but from a different council who were not included in the study. The interview guide for the FGDs is provided in Multimedia Appendix 3, and the interview guide for the KIIs is provided in Multimedia Appendix 4. The tools were initially prepared in English, then translated into Kiswahili and back-translated into English to ensure consistency. Kiswahili was used for all the interviews.

### Data Collection Procedures

#### Overview

We used 2 different data collection approaches (KIIs and FGDs) because of constraints related to geographical settings and participants’ availability. The KIIs were used to enable key leaders from regional and town or council health management teams to share their unique and sensitive perspectives. This approach facilitated the gathering of detailed insights in a confidential manner, as organizing FGDs was challenging for them due to the leadership roles of some participants; hence, bringing them together for FGDs was not easy. Since health care providers have common experiences with each other, the use of the FGDs’ interactive method revealed their group’s perspective as well as established norms for the group. The use of both KIIs and FGDs enabled the collection of complementary perspectives and is consistent with qualitative research recommendations on combining individual and group data collection approaches [28,29]. Participants recruited were individuals with direct experience with implementing and using the EHRS-ULTRA across different professional roles and health system levels, and the sample size was guided by the study objectives and the aim of obtaining rich, diverse, and information-rich accounts rather than by data saturation [30].

#### Key Informant Interviews

The KIIs were conducted by the principal investigator (GAO) in participants’ offices at private settings. Council health, town council health, and regional health management team members who participated in the EHRS-ULTRA pilot were invited to participate. All interviews were conducted and lasted approximately 20 minutes to 30 minutes each. Brief contextual notes were documented during and immediately after each interview to capture relevant observations, which supported the interpretations of the audio-recorded data. The interviews conducted were brief but effective due to the participants’ extensive experience in maternal and perinatal care and their direct involvement in the EHRS-ULTRA project, which led to a focused gathering of substantive and granular data. Sessions were audio-recorded via encrypted devices to guarantee data security and confidentiality.

#### Focus Group Discussions

We conducted 3 FGD sessions, each lasting 60 minutes to 90 minutes with a heterogeneous group composed of health care providers (clinicians, nurses, and medical attendants) selected from different levels of health facilities (dispensaries, health centers, and regional referral hospital) who were actively involved in the EHRS-ULTRA pilot implementation. One FGD was conducted with participants from 3 dispensaries, including 2 dispensaries from the Moshi district council and 1 from the Moshi municipal council. The second FGD involved participants from 2 health centers of the Moshi municipal council, and the third FGDs involved participants from a Mawenzi regional referral hospital. Each group consisted of 8 to 11 participants. This composition allowed the collection of diverse perspectives regarding system use across different professional roles and levels of maternal health care service delivery. All FGDs were conducted in private rooms within the respective participating health facilities. The discussions were moderated by the principal investigator (GAO) and assisted by a qualitative research expert (VWK), who documented key contextual observations and supported the moderation process. Each session began with a thorough explanation of the study’s objectives, followed by consent procedures. The participants were assigned unique identifiers for confidentiality. All discussions were conducted in Swahili and recorded via encrypted devices.

### Data Management and Analysis

The complete set of audio recordings (consisting of all the FGDs and KIIs) was transcribed verbatim in Kiswahili and translated into English by the research team before analysis. Transcripts were cross-checked against the corresponding audio recordings for accuracy and anonymized before analysis, and no repeated interviews were conducted or transcripts returned to participants for corrections. The analysis adopted an inductive orientation while remaining guided by the study objectives. Themes were developed through iterative, interpretive, and reflexive engagement with the dataset rather than being viewed as emerging directly from the data.

Data were analyzed using reflexive thematic analysis as described by Braun and Clarke [31,32], following 6 recursive phases: familiarization with the data, coding, generating initial themes, reviewing themes, defining and naming themes, and producing the report. The principal investigator (GAO) led the analysis by reading through the transcripts repeatedly to gain familiarity with the data before systematically coding them using NVivo software (Version 15), which was used to organize and manage codes rather than to quantify coding frequency or impose predetermined coding structure. During the initial stages of analysis, GAO and VWK collaboratively discussed and refined the coding approach while coding an initial subset of transcripts. GAO subsequently completed coding of the full dataset and led the iterative development, refinement, and interpretation of themes with ongoing reflexive discussions involving VWK and the wider research team. Throughout the analytic process, the research team engaged in regular analytical discussions to critically reflect on coding decisions, refine the developing themes, and support interpretation. Consistent with the principles of reflexive thematic analysis, no attempts were made to establish intercoder reliability or coder agreement.

### Reflexivity

Reflexivity was embedded throughout the research process. Researchers engaged in ongoing discussions to critically reflect on how their disciplinary backgrounds, professional experiences, and perspectives shaped data interpretation throughout the study process and theme development. Rather than seeking to eliminate researchers’ influence, reflexive engagement was used to enhance transparency and deeper interpretation of participants’ experiences. Reflective notes and regular analytical team discussions were maintained throughout to support ongoing critical reflection during coding and theme development.

### Trustworthiness

Trustworthiness was enhanced through multiple strategies. Credibility was strengthened through triangulation of data sources by combining findings from FGDs and KIIs and by including participants from different professional roles and levels of health care service delivery. Dependability was enhanced through detailed documentation of the research procedures, coding process, analytic decisions, and theme development. Research confirmability was improved by documenting personal reflections, maintaining clear records of research decisions and procedures, and engaging in regular peer discussions. This process ensured that interpretations remain aligned with participants’ accounts. Transferability was facilitated through detailed descriptions of the study setting, participant characteristics, and implementation context to enable readers to assess the applicability of the findings to similar contexts.

The codebook is available in Multimedia Appendix 5 and a public repository [33]. Reporting adhered to the COREQ (Consolidated Criteria for Reporting Qualitative Research) checklist [34] and is provided in Checklist 1.

### Ethical Considerations

Ethical approval was obtained from the Kilimanjaro Christian Medical Centre (KCMC) University Research Ethics Review Committee (number 2644) and the National Health Research Ethics Committee of the National Institute for Medical Research (number NIMR/HQ/R.8a/Vol. IX/4075). Permissions were also granted from the Office of the President of the Regional Administration and Local Government, Tanzania, Kilimanjaro Regional Administration, and local authorities.

Written informed consent was obtained from the participants in Swahili before the interviews, which included providing consent for audio recording and explaining the study objectives and their rights. Participation was voluntary, and confidentiality and anonymity were guaranteed. All the participants provided consent for their direct quotes to be used in this manuscript.

Participant confidentiality was ensured through private interview spaces and the removal of identifying information from all transcripts and reports. All audio recordings and transcripts were deidentified and stored in password-protected files with restricted access for the research team only. All direct identifiers were removed to ensure anonymity. KII participants (regional/town council/district level) are identified as KII-01 to KII-06, and FGD participants are identified as FGD1-R1 to FGD3-R11. Specific details on gender, profession, and facility names were excluded from quotations due to the small sample size. Participants were reimbursed for transportation costs, which were not intended as an inducement to join the study. This study was conducted in accordance with the Declaration of Helsinki.

## Results

### Demographic Characteristics of the Participants

A total of 35 participants were involved in the study, including 29 health care providers in FGDs and 6 members of regional, council, or town council health management teams in KIIs. Over one-half (18/35, 51%) of the participants were aged 25 years to 35 years. The majority were female (29/35, 83%), primarily from Moshi municipality (28/35, 80%), and were nurses (28/35, 80%). The participants’ characteristics are summarized in Table 1.

**Table 1.** Demographic and professional characteristics of participants involved in the electronich health record system (EHRS)-ULTRA implementation study in the Kilimanjaro region, Tanzania (n=35).

Characteristics	Participants, n (%)
Sex	
Male	6 (17)
Female	29 (83)
Age group (years)	
25‐35	18 (51)
36‐60	17 (49)
Districts	
Moshi District Council	7 (20)
Moshi Municipal Council	28 (80)
Health facility-affiliated participants (n=29)	
Chekeren Roman Catholic Dispensary	2 (7)
Himo Cogi Dispensary	3 (10)
Msaranga Dispensary	5 (17)
Majengo Health Center	5 (17)
Pasua Health Center	3 (10)
Mawenzi Regional Referral Hospital	11 (38)
Health facility–level participants^a^	
Dispensary	10 (35)
Health center	8 (28)
Regional referral hospital	11 (38)
Management-level participants^b^^,^^c^	
Council Health Management Team	2 (33)
Town-council Health Management Team	2 (33)
Regional Health Management Team	2 (33)
Professional category	
Nurse	28 (80)
Clinician	4 (11)
Medical attendant	3 (9)
Duration of using Electronic Health Record System-ULTRA	
<11 months	12 (34)
≥12 months	23 (66)

a Percentages calculated using n=29 as the denominator.

b Percentages calculated using n=6 as the denominator.

c Recruited from district, municipal, and regional health management teams.

### Description of the Themes

The findings of this study present an interpretive analysis of health care providers’ experiences and perceptions of using the EHRS_ULTRA for maternal and perinatal care. We developed 4 interrelated themes that reflect the perceived clinical and organizational value of the system, factors influencing its acceptance, and challenges affecting its sustainable implementation and future scalability. Themes and subthemes from the analysis are presented in Figure 1.

**Figure 1.**
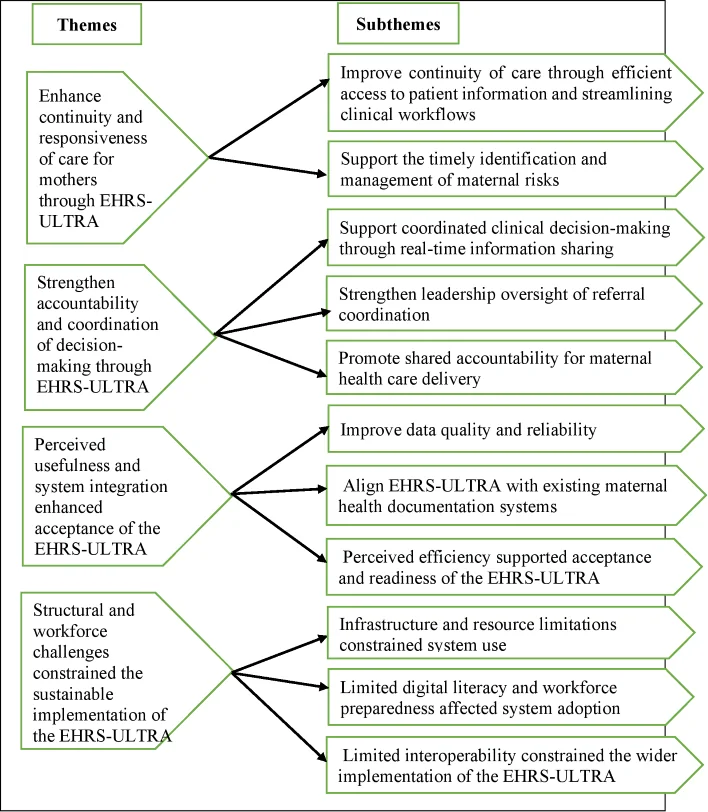
Illustrating themes and subthemes from key informants’ interviews and focus group discussions on user experiences with the electronic health record system (EHRS)-ULTRA.

### Theme 1: Enhance Continuity and Responsiveness of Care for Mothers Through EHRS-ULTRA

The theme shows how health care providers constructed EHRS-ULTRA as enhancing continuity and responsiveness of maternal and perinatal care. The provision of access to patient information, streamlining of clinical workflows, and timely identification of maternal risks were considered as essential methods that helped the system to deliver efficient, more coordinated, and more responsive service delivery.

#### Subtheme 1.1: Improve Continuity of Care Through Efficient Access to Patient Information and Streamlining Clinical Workflows

EHRS-ULTRA demonstrated the capacity to enhance continuity of maternal and perinatal care by enabling timely access to patient medical histories, referral information, and past medical records across levels of care. Streamlined retrieval of patient medical information and facilitated follow-up of mothers supported clinical decision-making and reduced the likelihood of missed continuation of care. In addition, the digital platform streamlined documentation processes by minimizing duplication of records, improving data storage and retrieval, and enabling providers to manage patient information more efficiently. Ultimately, the system served to simplify clinical workflows, enhance coordination among health care providers, and support timely service delivery, particularly in busy health care centers.

*The ULTRA system enables comprehensive patient management, allowing health care providers to review past treatments, such as the administration of magnesium to mothers with eclampsia, and track patient arrivals, facilitating continuity of care for both mothers and children.*

[FGD3-R1]

Participants viewed digital record storage system as providing the potential to create reliable and continuous care compared with paper-based documentation. Digital recording was perceived as the best method of safeguarding patient information and ensuring that clinical information can be accessible over time, as mentioned by one participant:

*Using a digital system allows for simultaneous storage of information without the risk of misplacing or losing papers, enabling easy traceability of data…*

[FGD3-R9]

The use of EHRS-ULTRA highlighted support for efficient managerial operations due to the users’ familiarity with this platform. The health managers explained that regular use of this system makes it much easier to get data compared with the use of traditional systems.

*Personally, the way we are used to the ULTRA system as a coordinator, if I want to review the information, I could do it easily.*

[KII-02]

#### Subtheme 1.2: Support Timely Identification and Management of Maternal Risks

EHRS-ULTRA supported the early identification and management of maternal risks through automated clinical alerts and increasing visibility of abnormal clinical findings. Digital notifications for conditions such as elevated blood pressure and low hemoglobin levels prompted timely clinical responses that were often constrained under paper-based documentation systems alone. These automated features facilitated earlier recognition of danger signs, enabling more timely interventions and enhancing the overall responsiveness of maternal and perinatal care:

*The system improves workflow by providing alerts for clients with low HB or high BP, prompting timely action. Unlike MTUHA, which only records values without alerts, the EHRS-ULTRA ensures that health care providers are notified of specific issues, facilitating better client care and service progression.*

[FGD1-R1]

Apart from identifying maternal risks at the point of care, participants also linked EHRS-ULTRA with timely access of information about mothers with danger signs, thereby enabling timely delivery of the required care. One participant described how this system intervenes on time:

*Generally, I can say the system is good; when mothers get to the clinic at the time of delivery to be able to know the danger signs, we were sometimes delayed […], but with the ULTRA system, it becomes easy to intervene on time and [...].*

[KII-01]

### Theme 2: Strengthen Accountability and Coordination of Decision-Making Through EHRS-ULTRA

This theme illustrates how EHRS-ULTRA functioned to strengthen accountability and coordination in maternal and perinatal care. Having real-time access to patient information enhanced referral monitoring, supported coordinated clinical decision-making, and promoted shared responsibility among health care providers and health system leaders.

#### Subtheme 2.1: Support Coordinated Clinical Decision-Making Through Real-Time Information Sharing

This subtheme illustrates how EHRS-ULTRA strengthened coordinated clinical decision-making by providing real-time access to patient information and referral status across health care facilities. The automated notifications enabled health care providers and managers to identify delays, monitor patient progress, and coordinate timely responses during maternal and perinatal emergencies. Rather than relying solely on paper-based communication, the digital system enhanced information sharing between facilities, thereby facilitating coordinated actions and more responsive referral management.

*The system allows leaders to pinpoint situations of unmet responsibility, especially concerning patient delays at facilities, and supports prompt follow-up by monitoring notifications and responses, enabling inquiries about actions taken during delays.*

[KII-02]

Receiving notifications of maternal and perinatal danger signs through EHRS-ULTRA offered the potential to improve timely service delivery and strengthen provider commitment particularly during referrals.

*[…] the pilot of a notification through EHRS-ULTRA for top leaders to monitor referrals could improve the situation by ensuring timely action and reducing negative experiences we used to get.*

[FGD2-R4]

#### Subtheme 2.2: Strengthen Leadership Oversight of Referral Coordination

EHRS-ULTRA enhanced leadership engagement in the referral process by enabling managers to track referrals and monitor patient progress in real time. This functionality allowed for timely interventions, enabled better support for frontline providers, and improved oversight of maternal and perinatal care. Additionally, the system increased accountability by providing decision-makers with visibility into delays and gaps in service delivery.

*The EHRS-ULTRA optimizes health care management by alerting leaders to new patient referrals with information on conditions and timing. It enhances readiness, monitors delays and referral statistics, and identifies facilities requiring mentorship, ultimately improving patient follow-up across facilities.*

[FGD3-R8]

#### Subtheme 2.3: Promote Shared Accountability for Maternal Health Care Delivery

EHRS-ULTRA enhanced shared responsibility among health care teams managing maternal and perinatal care. By facilitating access to referral information across various health care facilities, the platform fostered collective accountability for patient outcomes. This visibility prompted proactive engagement from providers and leadership to address gaps in health care service delivery.

*The system promotes collective accountability for patient care by ensuring that shared information reaches all responsible parties. This accountability fosters individual responsibility among health care providers and aids in addressing challenges at facility levels, such as resource shortages, by notifying leaders who can follow up on and resolve these issues.*

[FGD3-R7]

### Theme 3: Perceived Usefulness and System Integration Enhanced Acceptance of the EHRS-ULTRA

This theme illustrates the key factors that enhanced acceptance of EHRS-ULTRA during early implementation. Specifically, improvements in data quality, compatibility with existing maternal health documentation systems, and operational efficiency served as critical drivers of the system acceptability and potential for broader implementation.

#### Subtheme 3.1: Improve Data Quality and Reliability

This subtheme illustrates how EHRS-ULTRA enhanced the quality and reliability of maternal and perinatal health data by improving the accuracy, completeness, and readability of clinical documentation. Compared with paper-based records, the system functioned to reduce documentation errors and facilitate more reliable access to patient information, thereby supporting clinical workflow and decision-making at the facility level.

*This system has proven highly effective in capturing and reporting patient data, streamlining health care processes, and supporting accurate record-keeping, ultimately aiding better decision-making.*

[FGD3-R5]

Standardization of digital data entry within the EHRS-ULTRA was perceived to support improvement of document accuracy as well as providing security for the data. The platform guaranteed readability of the documents by addressing the legibility problems of the different writing styles in traditional records, thus minimizing documentation errors.

*ULTRA improves readability by accommodating various handwriting styles, reducing errors associated with traditional paper-based methods, and providing easy access to data.*

[FGD2-R1]

#### Subtheme 3.2: Align EHRS-ULTRA With Existing Maternal Health Documentation Systems

Compatibility with existing maternal health documentation systems, particularly the MTUHA registers, enhanced acceptance of EHRS-ULTRA by allowing providers to integrate the digital platform into routine workflows. However, absence of a few essential variables highlighted areas where further refinement is necessary to fully meet clinical documentation.

*The current system is aligned with the MTUHA books (numbers 6, 7, 12, and 13), which is satisfactory for now. However, if these books are updated in the future, they should also be consistent with the ULTRA system. Currently, many aspects are covered according to the MTUHA guidelines.*

[KII-04]

Although EHRS-ULTRA was perceived to improve routine workflows and obtained some of the metrics in maternal and children health, the inability to fill in all the immunization fields made it less efficient in the overall assessment for newborn care.

*The system was effective in simplifying work and included several variables relevant to maternal and child health, although it lacked BCG (Bacillus Calmette–Guérin) vaccination data, which we recommend be added for improved functionality.*

[FGD3-R3]

#### Subtheme 3.3: Perceived Efficiency Supported Acceptance and Readiness of the EHRS-ULTRA

EHRS-ULTRA functioned as a more efficient and cost-effective alternative to paper-based documentation systems. Significant reductions in printing costs (approximately €14 vs €1.64 [€1=US $1.11, as of October 31, 2026]), simplified reporting processes, and streamlined access to patient data served as critical operational advantages that increased confidence in the platform and strengthened support for its continued use and future implementation within maternal health care services.

*I advocate for the ongoing use of EHRS-ULTRA due to its cost-efficiency relative to traditional paper methods. Printing one MTUHA book costs around 40,000 Tanzanian shillings, with possible additional purchases needed within three months, hindering availability. Conversely, EHRS-ULTRA requires merely a minimal monthly internet bundle of about 5,000 Tanzanian shillings, greatly lowering printing costs.*

[FGD1-R9]

### Theme 4: Structural and Workforce Challenges Constrained Sustainable Implementation of the EHRS-ULTRA

This theme examines the structural, technical, and workforce challenges that constrained implementation of the EHRS-ULTRA. Specifically, infrastructure limitations, inadequate digital competencies, and interoperability challenges served as major barriers to sustaining and scaling the platform within routine maternal and perinatal health care.

#### Subtheme 4.1: Infrastructure and Resource Limitations Constrained System Use

Inadequate infrastructure served as a major barrier to the routine use of the EHRS-ULTRA. Frequent electricity interruptions, unreliable internet connectivity, and insufficient digital devices disrupted clinical workflows, forcing providers to revert to paper-based documentation and reducing confidence in the system’s reliability for routine maternal and perinatal care.

*The main challenges identified are internet connectivity, power outages, and funding. The offline and online nature of the EHRS-ULTRA poses risks of data loss if synchronization fails when devices lack power and internet access […].*

[KII-06]

Insufficient hardware and low ratio of devices to clinical practitioners posed a major structural obstacle to the continuous utilization of EHRS-ULTRA technology. The inconsistent supply of tablets regularly disrupted the clinical routine, which necessitated the use of the system.

*Insufficient tablets at work might affect EHRS-ULTRA utilization, with only 6 workers provided with 6 tablets. If only 3 tablets are operational on a given day, other providers might face disruptions in their workflow. Ensuring enough tablets for all sections is essential to lessen reliance on paper.*

[FGD3-R3]

#### Subtheme 4.2: Limited Digital Literacy and Workforce Preparedness Affected System Adoption

Varying levels of digital literacy and proficiency with electronic health systems directly impacted the regular use of EHRS-ULTRA across facilities. Consequently, continuous training, mentorship, and supportive supervision are essential to build user confidence, improve data quality, and ensure long-term system sustainability. Furthermore, extending digital health training to community health workers offers significant potential to strengthen the continuity of care between communities and health facilities.

*To ensure effective system operation, thorough training is essential. Given varying levels of computer experience, regular training sessions and monthly follow-ups at different facilities are crucial to identify and address challenges for optimal performance.*

[FGD3-R1]

Training on digital health tools should go beyond facility-based health care professionals to include community health care workers, given their active involvement in frontline health care service delivery.

*[…] CHWs are essential in managing nutrition and monitoring at-risk pregnant women in the community. Equipping them with the EHRS-ULTRA package can improve their capacity to provide thorough feedback to health care providers […].*

[KII-03]

#### Subtheme 4.3: Limited Interoperability Constrained Wider Implementation of the EHRS-ULTRA

Limited interoperability with other health information systems represented a primary obstacle to the wider implementation of EHRS-ULTRA. Inadequate integration across digital platforms resulted in fragmented referral information, duplication of data entry, and challenges with tracking the patient’s journey across facilities. Consequently, gradual implementation, sustainable financing, and coordinated national planning are necessary to achieve successful scaling:

*Integrating the eHMS (electronic health management system) with EHRS-ULTRA is recommended for effective patient tracking, covering tests ordered, labor progression, delivery details, and vaccinations.*

[FGD3-R6]

In order to avoid significant disruptions and facilitate successful scaling, participants recommended a controlled, phased rollout strategy and highlighted the necessity of time-limited electronic pilot programs to test system functionality prior to large-scale implementation, citing lessons learned from prior government digital health initiatives.

*[...] to evaluate the EHRS-ULTRA system, a pilot study is proposed at the Regional Referral Hospital for one to three months with no paper use. This will test two to three hospitals and assess results for improvements before nationwide implementation, similar to the gradual rollout of the M-Mama system.*

[FGD3-R7]

Scaling up EHRS-ULTRA faces significant challenges without dedicated budget allocations for ongoing running costs, which has the potential to compromise its full functionality.

*[…] if scalability will be done without allocating funds for the internet, it will cause challenges, but if we allocate on a financial year, we know that by July, we start with the system at all facilities after budgeting in our plan […].*

[KII-04]

## Discussion

### Principal Findings

This study explored health care providers’ experiences and perceptions of using the EHRS-ULTRA for maternal and perinatal care during its pilot implementation in health facilities in the Kilimanjaro region. Participants described the perceived clinical and organizational benefits of the system, factors that supported its acceptance, and structural and workforce challenges that may influence its future implementation and scalability. These findings provide practical insights into the opportunities and challenges of implementing digital maternal health systems within routine health care and may help guide future implementation in similar low-resource settings. In addition, these findings support the WHO recommendations on digital health interventions aimed at strengthening health systems, particularly in low-resource settings, focusing on improving continuity of care, data use, workflow integration, and health workforce support [14-16].

### Perceived Benefits and Alignment With the Clinical Workflow

Participants perceived that the EHRS-ULTRA improved data quality, timeliness, and access to information across different levels of health care. Compared with paper-based MTUHA registers, participants perceived the EHRS-ULTRA to support better documentation, facilitate continuity of care, and improve maternal and perinatal data management within and across health facilities participating in the pilot. Despite these benefits, participants noted that infrastructural limitations and the continued parallel use of paper systems could reduce usability and risk underutilization of the system. Participants further expressed concern that maintaining multiple documentation systems could become burdensome, reducing end user ownership and the sustained use of the EHRS-ULTRA. These results are consistent with studies from Kenya and Ghana, which reported that the use of EHRSs improves the consistency, completeness, correctness, and reliability of clinical data [35,36]. However, these findings contrast with studies from Mtwara (Tanzania), Bangladesh, and Rwanda, where users still struggle to address systemic challenges, including the increased workload and reduced efficiency in recording accurate maternal and perinatal information [37-39]. These results reinforce the WHO recommendations that effective EHRS use, supported by appropriate infrastructure and workforce capacity, can enhance care continuity, improve clinical decision-making, and optimize the use of health data [15,16].

In addition, the EHRS-ULTRA was perceived to support data-driven decision-making to facility leaders, health managers, and policymakers by providing real-time data that enables them to assess health care delivery, identify gaps in care, and monitor daily operations and patient flow more effectively. Real-time data also helped identify operational challenges encountered by health care providers, such as missed follow-up, referral delays, and workflow disorganization, thereby supporting more targeted resource allocation through continuous monitoring of care. These findings are consistent with studies from Tanzania and South Africa, which have shown that the use of EHRSs can improve health data reliability and evidence-based decision-making and strengthen health system monitoring of maternal and perinatal care [40,41].

Overall, participants perceived that the EHRS-ULTRA improved data capture and supported clinical decision-making while strengthening accountability among health care providers and leadership in maternal and perinatal health services.

Furthermore, participants highlighted that the EHRS-ULTRA improved coordination and shared accountability across different levels of maternal and perinatal care. The real-time visibility of referrals and notifications was viewed to enhance responsiveness among both health care providers and leadership during maternal emergencies. Therefore, these results suggest that digital referral tracking could strengthen coordination and transparency in the delivery of maternal health care in resource-constrained environments.

### Key Enablers of EHRS-ULTRA Adoption

Leadership engagement and involvement were perceived as vital enablers of EHRS-ULTRA adoption. The active engagement of leaders in the EHRS initiative and data-driven reviews was perceived to increase accountability, awareness, commitment, and teamwork among staff. Participants also noted that engagement of health administrative leaders during a pilot was supportive and strengthened system usability and staff buy-in. These findings highlight the importance of engaging informed leaders throughout the implementation process. Similarly, studies from Ghana and Uganda highlighted that engagement of informed leaders strengthens ownership and supports the long-term sustainability of EHRS implementation [42,43]. In addition, EHRS adoption is enhanced by supportive leadership by fostering team accountability, creativity, and motivation [44-47]. Similarly, the WHO Global Strategy on digital health emphasizes effective governance, stakeholder engagement, and workforce preparation [14].

Finally, participants perceived the cost-effectiveness of EHRS-ULTRA as an important enabler of future adoption. Participants perceived that EHRS-ULTRA reduced administrative burden, including paper-based documentation and printing costs, thereby increasing its potential for adoption. This aligns with findings from other studies, whereby implementing an EHRS leads to substantial savings, such as elimination of physical storage space needs, staff focus on patient care, reduction in expenditure on paper and administrative costs, and faster workflows [48,49]. In contrast, other studies reported that EHRS implementation did not reduce billing costs and that customization increased costs [49-51]. Generally, these findings suggest that implementing digital health care technologies such as EHRS-ULTRA requires more than technical solutions alone. Active leadership support for data-driven practices is essential for successful implementation, which is consistent with the broader findings of our recent systematic review [17].

### Organizational and Contextual Barriers to Adoption

In addition to the perceived benefits, participants identified several significant barriers to successful implementation of the system, including inadequate digital infrastructure, unstable electricity supply, limited access to high-speed internet, and insufficient digital devices. These findings are consistent with evidence showing that infrastructure challenges, such as unreliable electricity, poor internet, and insufficient devices, are a significant barrier to the effective implementation of EHRSs in LMIC [18,52,53]. Participants also reported that limited IT support and aspects of the system design, including restricted editing privileges and challenges with tracking clients across facilities, reduced their confidence in the system and slowed its adoption. Similarly, studies from SSA and the Philippines reported that IT support can hinder effective use of the EHRS in low-resource settings [54,55]. Consistent with our systematic review, these findings suggest that users’ acceptance and ongoing implementation support are critical determinants of successful EHRS adoption, often outweighing the system’s technical capabilities alone [17].

### Readiness for Scale-Up and Policy Considerations

Participants perceived that phased implementation, targeted financial planning, improved interoperability with coexisting digital platforms, and coordinated policy support would be necessary for the national scale-up of EHRS-ULTRA. Participants identified fragmented digital health strategies, limited system integration with existing systems such as the electronic health management system and M-Mama, and uncertain funding as major risks to the sustainability of the EHRS-ULTRA over the long term. These findings are consistent with previous studies from Uganda, Rwanda, India, and Tanzania, where policy misalignment and funding constraints limited the expansion of EHRSs [6,56-59]. Participants recommended a phased implementation approach supported by coordinated government, harmonized policies, and sustainable financial support to facilitate effective system integration and provide opportunities for shared learning, before nationwide scale-up [6,56-59].

### Implications for Implementation

Although our results highlight the importance of system features for successful implementation of the EHRS-ULTRA, they also indicate that successful implementation requires a supportive environment characterized by reliable and stable infrastructure, responsive health leadership, access to reliable technical assistance, and integration of digital protocols into routine clinical workflows. Successful digital health interventions require interoperable systems, supportive governance structures, reliable infrastructure, ongoing technical support, and integration into routine service delivery [14-16].

Addressing these elements may strengthen data-informed decision-making, governance, and accountability for maternal and perinatal health, with broader benefit for health services delivery. Without addressing these interrelated factors, large-scale digital transformation in health care is likely to remain challenging, consistent with the broader conclusions of our systematic review on digital maternal and perinatal health technologies [17].

### Strengths and Limitations

A major strength of this study is that it included participants from multiple levels of the health system, providing a comprehensive understanding of experiences with the EHRS-ULTRA. However, this study was conducted within a pilot implementation in one geographical area of Tanzania, which may limit the transferability of the findings to other settings. Moreover, the research findings should be considered alongside existing paper and digital documentation systems, as user perspectives were gathered during an early pilot implementation stage.

Additionally, because the study relied on self-reported experiences, social desirability bias cannot be excluded. However, this was mitigated by including participants from different facility levels and professional roles and by triangulating findings from FGDs and KIIs. The study largely focused on user perspective and acceptability rather than system performance evaluation, data quality, and clinical outcomes, which have been examined in previous pilot studies.

### Conclusion

Health care providers and health managers perceived the EHRS-ULTRA as an acceptable and useful tool for enhancing maternal and perinatal health care documentation, ensuring continuity of care, and supporting clinical workflows. Key success factors for its implementation include leadership engagement, workflow integration, technical support, and the perceived value of the system in supporting clinical decision-making. These findings align with the WHO’s recommendations for enhancing maternal and perinatal care in low-resource settings. These findings are also consistent with our systematic review, which highlighted that user acceptance and ongoing implementation support are critical determinants of EHRS success, often outweighing technical capabilities alone [17].

However, barriers including inadequate infrastructure, limited digital literacy, and challenges associated with transitioning from paper-based systems may hinder wider implementation. Addressing these barriers will require sustained investments in workforce training, digital infrastructure, supportive supervision, continuous system improvement, and policy refinement. Future research should include quantitative evaluations of the EHRS-ULTRA to assess system performance, data quality, and its impacts on maternal and perinatal health outcomes.

## Supplementary material

10.2196/90400 Multimedia Appendix 1 Workflow from patient registration to reporting and feedback within the electronic health record system (EHRS)-ULTRA platform.

10.2196/90400 Multimedia Appendix 2 Electronic health record system (EHRS)-ULTRA reporting and supervision workflow chart.

10.2196/90400 Multimedia Appendix 3 Interview guide for the focus group discussions (FGDs).

10.2196/90400 Multimedia Appendix 4 Interview guide for the key informant interviews (KIIs).

10.2196/90400 Multimedia Appendix 5 Code book.

10.2196/90400 Checklist 1 COREQ (Consolidated Criteria for Reporting Qualitative Research) checklist for electronic health record system (EHRS)-ULTRA.

## Abbreviations

COREQ: Consolidated Criteria for Reporting Qualitative Research
DHIS2: District Health Information System 2
EHRS: electronic health record system
FGD: focus group discussion
KCMC: Kilimanjaro Christian Medical Centre
KII: key informant interview
LMIC: low- and middle-income countries
mHealth: mobile health
MTUHA: Mfumo wa Taarifa za Uendeshaji wa Huduma za Afya
SSA: sub-Saharan Africa
WHO: World Health Organization
