# Supplementary material for: Health Care Providers’ Experiences and Perspectives on the Use of Electronic Health Record System-ULTRA for Maternal and Perinatal Care in Kilimanjaro, Tanzania: Qualitative Study
Source: JMIR Form Res. 2026 Nov 6;10:e90400. doi: 10.2196/90400 (PMC13743068; doi:10.2196/90400)
Supplement: Supplementary file 1 [file formative-v10-e90400-s001.docx]

**Multimedia Appendix 1:** The workflow from patient registration to reporting and feedback within the EHRS-ULTRA platform

**Figure 2. The workflow from patient registration to reporting and feedback within the EHRS-ULTRA platform**

**Note:**

*The diagram illustrates the flow of clinical data from healthcare providers (ANC, L&D, and PNC) through data entry into the system using tablets or Android smartphones, and then processing and automatic reporting generation within EHRS-ULTRA in a DHIS2-based system. The data is then shared to facility level review (in-charges & providers), and individual/aggregated reporting is sent to authorities for close monitoring and follow-up for system improvement. Additionally, it depicts feedback mechanisms, facility-level reviews, and health authority reviews during the pilot deployment phase in Kilimanjaro, Tanzania. The goal of the review procedure was to offer oversight and comments to enhance the system as a whole.*
