# Supplementary material for: Health Care Providers’ Experiences and Perspectives on the Use of Electronic Health Record System-ULTRA for Maternal and Perinatal Care in Kilimanjaro, Tanzania: Qualitative Study
Source: JMIR Form Res. 2026 Nov 6;10:e90400. doi: 10.2196/90400 (PMC13743068; doi:10.2196/90400)
Supplement: Supplementary file 2 [file formative-v10-e90400-s002.docx]

**Multimedia Appendix 2:** EHRS-ULTRA reporting and supervision workflow chart

Healthcare providers (ANC/L&D/PNC staff)

Head of RCH & Facility In-charges (Supervision & validation)

Regional and Town/District Council Health Management Teams (RHMT/CHMT/THMT)

*Leadership engagement and supervision supported system use, monitoring, and referral tracking during the pilot phase.*

**Figure 3. EHRS-ULTRA reporting and supervision workflow chart**

**Note:**

*The EHRS-ULTRA pilot implementation's reporting and supervision structure is depicted in this diagram, which shows hierarchical data, reporting mechanism flow from healthcare providers directly to the ULTRA project team for technical support, and then back to healthcare providers, the head of RCH, and facility in-charges for system validation and supervision. Once more, the ULTRA project team reports to regional health management teams and town/district councils for administrative assistance. During the deployment of EHRS-ULTRA in Kilimanjaro, Tanzania, the figure illustrates leadership engagement and feedback systems.*
