# Supplementary material for: Health Care Providers’ Experiences and Perspectives on the Use of Electronic Health Record System-ULTRA for Maternal and Perinatal Care in Kilimanjaro, Tanzania: Qualitative Study
Source: JMIR Form Res. 2026 Nov 6;10:e90400. doi: 10.2196/90400 (PMC13743068; doi:10.2196/90400)
Supplement: Supplementary file 4 [file formative-v10-e90400-s004.docx]

**Multimedia Appendix 4: Interview Guide for KIIs**

Key informant interview (KII) guide for exploring Healthcare Providers’ Experiences and Perspectives on the Use of Electronic Health Record System-ULTRA for Maternal and Perinatal Care in Kilimanjaro, Tanzania.

*This document is used to guide the gathering of information from participants who have been serviced in the health facilities of Kilimanjaro. The form should be read, and questions should be answered in a language in which the participant is fluent. Information is being gathered on the available electronic health data recording systems at each level of the maternal health care system in Kilimanjaro region.*

## Section A: Background Information on KII.

**KII Identification number:**

**Region**: …………………………...	**District**: ……………………….

**Date:**

**Start time:**

**Finish time**

**Duration of KII:**

**Description of area where interview took place:**

**Facilitator/moderator’s – name:**

**Note taker– name:**

| **Participant’s assigned number** | **Sex** | **Age** | **Duration of using EHRS-ULTRA** | **professional** | **Facility Name** | **Did you receive EHRS-ULTRA training?** |
| --- | --- | --- | --- | --- | --- | --- |
| **1** |  |  |  |  |  |  |

**Introduction to the participant of the KII by researcher, welcome and thanks.**

- *Inform the participants that the conversation will be recorded for purpose of the study only*
- *Check if participants have any questions, provide answers. Ask interviewees to sign the study consent form.*

**Start recording.**

1. Can you please explain to me in details regarding your experience in using the EHRS- ULTRA in the past months?

**Probe:**

- How did you feel about using EHRS-ULTRA, do you think EHRS-ULTRA should be simple to use, should we remove something? quality and consistency of the data collected through ULTRA, what should we add if any, what was the most challenging aspect of using ULTRA,

2. In your view, can you tell us at what time is reasonable to start using EHRS-ULTRA and stop using paper-record (MTUHA) system and why?

**Probe:**

- Is it the right time now or up to when and why? do you prefer to use only ULTRA system or even any other electronic record systems and why?
- if we start implementing ULTRA what are the foreseeable barriers and facilitators identified during the ULTRA system implementation

3. Would you tell us if there is any technical support you would need for you to be able to use the ULTRA system and why the support is needed?

**Probe:**

- what type of support, at what stages and how frequent will the support be needed,
- after providing the support mentioned, tell us your opinion of scaling up the ULTRA system be adapted and scaled up all over the country*.*

4. Would you please share with us your experience and opinions about how does the ULTRA system help on referral system?

**Probe:**

- The referral system has improved good continuum of care, does not need to use paper any more,
- the benefits of the referral system,
- the differences between the new and old referral systems,
- challenges of using the referral system,
- your advice to policymakers and health authorities regarding the usage of the ULTRA system particularly with regard to referral system use.

5. Can you share your experience on how does the ULTRA system help on notification of danger signs of maternal and perinatal?

**Probe:**

- the benefits of the notification systems, the differences between the normal practice and the new system of notification practice,
- what are the challenges, and your advice to policymakers and health authorities regarding the usage of the ULTRA system, particularly with regard to notification system use.

*Check if the interviewee has any questions or would like to add information which wasn’t asked for in the session.*

Thank you for your participation.

THE END
