# Supplementary material for: Health Care Providers’ Experiences and Perspectives on the Use of Electronic Health Record System-ULTRA for Maternal and Perinatal Care in Kilimanjaro, Tanzania: Qualitative Study
Source: JMIR Form Res. 2026 Nov 6;10:e90400. doi: 10.2196/90400 (PMC13743068; doi:10.2196/90400)
Supplement: Supplementary file 5 [file formative-v10-e90400-s006.docx]

**Multimedia Appendix 6: COREQ checklist for EHRS-ULTRA**

**Domain 1: Research Team and Reflexivity**

| **No.** | **Item** | **Description (Your Study)** | **Pg. No. Lines** |
| --- | --- | --- | --- |
| **1** | Interviewer/facilitator | Principal Investigator (GAO) conducted all KIIs and facilitated FGDs. | P.9. Lines 187-190 |
| **2** | Credentials | GAO, MPH and PhD candidate; VK, PhD candidate, qualitative research expert, and social worker. | P.9. Lines 187-192 |
| **3** | Occupation | GAO was a PhD candidate and public health researcher with experience in maternal health and qualitative research. VK was a PhD candidate, qualitative researcher, and social worker.  . | P.9. Lines 187-192 |
| **4** | Gender | The research team included both female and male researchers. | P.9. Lines 187-190 |
| **5** | Experience and training | Both researchers had prior training and experience in qualitative health systems research and interview facilitation. | P.9. Lines 187-192 |
| **6** | Relationship established | Participants were informed about the study before data collection. Although some participants were familiar with the study team through the EHRS-ULTRA implementation activities, there were no direct supervisory relationships between investigators and participants. | P.9. Lines 192-195 |
| **7** | Participant's knowledge of the interviewer | Participants were informed about the study objectives, the interviewer’s role, and the voluntary and confidential nature of participation before data collection. | P.8-9,11. Lines 183-185, 244-247 |
| **8** | Interviewer characteristics | The research team engaged in ongoing reflexive discussions throughout data collection and analysis to critically consider how their disciplinary backgrounds, professional experiences, and perspectives shaped data interpretation. | P.8,12-13. Lines 195-197, 269-272 |

**Domain 2: Study Design**

| **No.** | **Item** | **Description (Your Study)** | **Pg. No. Lines** |
| --- | --- | --- | --- |
| **9** | Methodological orientation | Qualitative exploratory descriptive design; Reflexive Thematic Analysis following Braun and Clarke. | P.6,12. Lines 134,259 |
| **10** | Sampling | Purposive sampling was used to recruit healthcare providers and health managers involved in the pilot implementation. | P.8. Lines 180-183 |
| **11** | Method of approach | Eligible participants were identified through facility management teams and invited in person by the research team. | P.8-9. Lines 181-186 |
| **12** | Sample size | Total N= 35; Six KIIs and three FGDs with 8–11 participants each. | P.9,10,11, 15. Lines 224-226, 234,241,316-317 |
| **13** | Non-participation | No invited participants declined to participate, and no withdrawals occurred after recruitment. | P.9. Lines 185-186 |
| **14** | Setting of data collection | Conducted in private rooms/offices within participants’ workplaces/facilities. | P.10,11. Lines 224-225,243-244 |
| **15** | Presence of non-participants | KIIs conducted in private settings; FGDs in Private rooms (no non- participants present). | P.9,10,11,13. Lines 185-186,224-225,243-244, 308-309 |
| **16** | Description of sample | Participants included healthcare providers working in ANC, L&D, PNC, together with facility manager (in-charges), CHMT, THMT, and RHMT members. Table 1 provides full demographic details | P.8,15-16. Lines 173-178, 317-361 |
| **17** | Interview guide | Semi-structured interview guides based on literature and expert input, translated into Kiswahili, and pilot-tested before data collection. | P. 9-10. Lines 202-207 |
| **18** | Repeat interviews | Repeat interviews were not conducted. | P.12. Lines 254-255 |
| **19** | Audio/visual recording | All sessions were audio-recorded using encrypted digital devices with participants' consent | P.11,12. Lines 231,248-249 |
| **20** | Field notes | Brief contextual notes documented during and immediately after sessions to capture observations from the data collection process and support interpretation of the audio-recorded data. | P.11. Lines 227-228, 244-246 |
| **21** | Duration | KIIs lasted 20–30 minutes; FGDs lasted approximately 60–90 minutes. | P.10-11,10. Lines 226-227, 234 |
| **22** | Data saturation | Data saturation was not used as a sample size criterion, consistent with Reflexive Thematic Analysis. Participant recruitment was guided by the study objectives and the richness and diversity of participants’ experiences. | P.10. Lines 219-222 |
| **23** | Transcripts returned | Transcripts were not returned to participants for comment or correction. | P.12. Lines 252-256 |

**Domain 3: Analysis and Findings**

| **No.** | **Item** | **Description (Your Study)** | **Pg. No. Lines** |
| --- | --- | --- | --- |
| **24** | Number of data coders | Initial coding done collaboratively by GAO and VK; full coding led by GAO with ongoing reflexive discussions and analytical reviews with VK and research team. | P.12-13. Lines 265-272 |
| **25** | Description of coding tree | Codes developed iteratively and refined. The codebook available in Multimedia Appendix 5. | P.12-14. Lines 260-272, 292-293 |
| **26** | Derivation of themes | **Themes developed through an inductive, iterative, interpretive, and reflexive process following Braun & Clarke.** | P.12. Lines 256-261 |
| **27** | Software | NVivo version 15 was used for data management and coding. | P.12. Lines 261-265 |
| **28** | Participant checking | Participant validation (member checking) was not conducted. | P.12-13. Lines 253-256, 286-291 |
| **29** | Quotations presented | Representative participant quotes presented in results under each subtheme. | P.19-28. Lines 425-428,433-434,438-439, etc. |
| **30** | Data and findings consistent | Themes supported by multiple data sources (KIIs and FGDs). | P.13,17. Lines 282-284,366-371 |
| **31** | Clarity of major themes | Four major themes clearly identified and illustrated in Figure 1 and main text headings (results- description of theme): Enhance continuity and responsiveness of care for mothers through EHRS-ULTRA; Strengthen accountability and coordination of decision-making through EHRS-ULTRA; Increase acceptance of EHRS-ULTRA through perceived usefulness and system integration; Overcome structural and workforce barriers to sustainable implementation of EHRS-ULTRA. | P.17-19, 21,23,25. Lines 407,457,504,552-553 |
| **32** | Clarity of minor themes | **Subthemes clearly delineated under each major theme heading (Subthemes 1.1-1.2, 2.1-2.3, 3.1-3.3 and 4.1-4.3).** | P. 18-27. Lines 401-591. |
